# Assessing the Influence of Vegan, Vegetarian and Omnivore Oriented Westernized Dietary Styles on Human Gut Microbiota: A Cross Sectional Study

**DOI:** 10.3389/fmicb.2018.00317

**Published:** 2018-03-05

**Authors:** Carmen Losasso, Ester M. Eckert, Eleonora Mastrorilli, Jorg Villiger, Marzia Mancin, Ilaria Patuzzi, Andrea Di Cesare, Veronica Cibin, Federica Barrucci, Jakob Pernthaler, Gianluca Corno, Antonia Ricci

**Affiliations:** ^1^Department of Food Safety, Istituto Zooprofilattico Sperimentale delle Venezie, Legnaro, Italy; ^2^Microbial Ecology Group, Institute of Ecosystem Study, National Research Council, Verbania, Italy; ^3^Limnological Station, Department of Plant and Microbial Biology, University of Zurich, Zurich, Switzerland; ^4^Department of Information Engineering, University of Padova, Padova, Itay; ^5^Dipartimento di Scienze della Terra, dell’Ambiente e della Vita, University of Genova, Genova, Italy

**Keywords:** gut microbiota, feeding type, nutritional intake

## Abstract

Diet and lifestyle have a strong influence on gut microbiota, which in turn has important implications on a variety of health-related aspects. Despite great advances in the field, it remains unclear to which extent the composition of the gut microbiota is modulated by the intake of animal derived products, compared to a vegetable based diet. Here the specific impact of vegan, vegetarian, and omnivore feeding type on the composition of gut microbiota of 101 adults was investigated among groups homogeneous for variables known to have a role in modulating gut microbial composition such as age, anthropometric variables, ethnicity, and geographic area. The results displayed a picture where the three different dietetic profiles could be well distinguished on the basis of participant’s dietetic regimen. Regarding the gut microbiota; vegetarians had a significantly greater richness compared to omnivorous. Moreover, counts of Bacteroidetes related operational taxonomic units (OTUs) were greater in vegans and vegetarians compared to omnivores. Interestingly considering the whole bacterial community composition the three cohorts were unexpectedly similar, which is probably due to their common intake in terms of nutrients rather than food, e.g., high fat content and reduced protein and carbohydrate intake. This finding suggests that fundamental nutritional choices such as vegan, vegetarian, or omnivore do influence the microbiota but do not allow to infer conclusions on gut microbial composition, and suggested the possibility for a preferential impact of other variables, probably related to the general life style on shaping human gut microbial community in spite of dietary influence. Consequently, research were individuals are categorized on the basis of their claimed feeding types is of limited use for scientific studies, since it appears to be oversimplified.

## Introduction

The human intestine is a complex biological network that accounts for a great variety of microorganisms. Because this microbial community is known to have a profound impact on many aspects of human health including the immune system (Cerf-Bensussan and Gaboriau-Routhiau, 2010), inflammatory disease and obesity (Turnbaugh et al., 2006), a number of studies on the diversity of the bacterial populations of the human gut have been carried out in recent years (Turnbaugh et al., 2006; McDonald et al., 2015; Davenport et al., 2017; Moran-Ramos et al., 2017). Diet and lifestyle strongly profile the gut microbiota (i.e., the entire gut microbial community) (De Filippo et al., 2010; De Filippis et al., 2016) and dietary modifications can provoke changes in the relative abundances of microbial taxa. Moreover, the human gut microbiota dynamically interacts with the external environment in a bidirectional manner as bacteria can move between ecosystems: from animals to humans, through manure and feces, to water and soil and return to humans and animals by food and feed (Schjørring and Krogfelt, 2011). Thus, both, the environmental context and food sources exert a pivotal influence on the composition and diversity of the gut microbiota of individuals and whole populations.

In this context, the vegetarian diets including vegan, have obtained recognition as healthy and potentially therapeutic feeding types. If appropriately planned these diets may provide health benefits for the prevention and treatment of certain diseases including ischemic heart disease, type 2 diabetes, hypertension, certain types of cancer, and obesity (Melina et al., 2016). The possibility that any such health advantage might be linked to a unique protective gut microbiota profile has been the object of previous studies (Glick-Bauer and Yeh, 2014).

To describe the differences in the microbiota profile deriving from different food choices, Wu et al. (2011) found that three enterotypes could be associated with different dietary profiles: the genus *Prevotella* was found to be adapted to a carbohydrate-dominated metabolism and a vegetarian diet; *Bacteroides*, in turn, was linked to diets that were high in protein and animal derived products (mostly omnivorous) and microbiota rich in *Firmicutes* (which includes the enterotype *Ruminococcus*) was strongly associated with a fat based *westernized* diet (De Filippo et al., 2010) and obesity (Ley et al., 2006), even though it seems that species of the same taxonomic group may harbor different metabolic characteristics (De Filippis et al., 2016). On the other hand, negligible differences were found between the gut microbiota of two cohorts of vegans and omnivores in the United States (Wu et al., 2016).

Thus, to date, the available information on the human gut microbiota does not allow inference of the probability that a single individual (or a group) will belong to a specific dietary type, such as vegetarian or omnivorous, from the composition of their gut microbiota. Moreover, detailed comparative studies of humans from the same society with different dietary preferences are still scarce, and that also consider features such as the nutritional status, the daily food frequencies and the total composition of the meal and their cumulative impacts.

In order to fill this gap in knowledge, the gut microbiota composition of three groups following vegan, i.e., free of any animal derived product, vegetarian or omnivorous diets was investigated and variables such as anthropometric parameters and qualitative and detailed quantitative dietary information, known to exert a considerable impact on modulating gut microbiota (Derrien and Veiga, 2017) were used to examine possible differences between the studied groups.

## Materials and Methods

### Participant Recruitment and Metadata Collection

Between August and December 2013, 101 individuals categorized as vegans (VG) (*N* = 26), vegetarians (V) (*N* = 32), and omnivores (O) (*N* = 43), were recruited on a voluntary basis and informed written consent was signed by each participant VG and V were enrolled with the collaboration of the Italian Society of Vegetarian Nutrition^1^ and the Italian League against Vivisection^2^, while O participants were recruited with the collaboration of the Regional Service for Food Hygiene and Nutrition Promotion. All participants were enrolled using the following inclusion criteria: being strictly vegan, vegetarian, or omnivorous for more than 12 months prior to the study; being adults; currently not taking antibiotics and not having taken antibiotics in the last 12 months; being non-smokers and not having smoked in the last 12 months; not having been hospitalized for at least 24 months prior to the study; not being prescribed medical drugs; not having intestinal (Crohn’s disease, chronic ulcerative colitis, bacterial overgrowth syndrome, constipation, celiac disease, Irritable Bowel Syndrome) or other pathologies (type I or type II diabetes, cardiovascular or cerebrovascular diseases, cancer, neurodegenerative disease, rheumatoid arthritis, allergies); not being pregnant or lactating.

All participants were checked for their nutritional status (overweight, normal weight, or underweight), both calculating the body mass index (BMI) and deriving the total body fat mass (BFM), total body lean mass (BLM), and total body water (BWM), by multifrequency bioimpedance analysis (MFBIA) using a Tanita MC180MA device, then amending possible biases as suggested by Ellegård et al. (2016). The final sample was composed of participants fulfilling homogeneity criteria for age, BMI, BFM, and BLM parameters.

Quantitative and qualitative data on habitual dietary intake was assayed using a semi-quantitative food frequency questionnaire (FFQ), spanning 14 days of observation before the fecal sample was delivered. Moreover, a results reliability test was applied by means of a 24 h dietary recall (24HR) administered to each participant, as recommended by National Cancer Institute (United States)^3^. Additional questions about consumption of animal products were asked of each participant in order to understand if their dietary habits in the last year diverged from the self-declared diet type.

FFQ data were then analyzed using WinFood^®^ software (Medimatica Srl, Italy) in order to extract a table of 76 macro- and micro-nutrients in the dietary compositions among all the participants. Then, average consumption of all 76 nutrients over the observed fortnight was considered in all analyses. Secondly, dietary habit information was summarized by describing the average percentage of total caloric intake imputed to the three macronutrient categories of lipids, proteins, and carbohydrates. Both the complete and reduced nutritional data were used in subsequent analyses. Informed consent for specimen acquisition and data processing was acquired.

### Fecal Sample Collection and DNA Extraction

Each donor was provided with a pair of sterile gloves and two sterile containers (one Coprotainer^®^ Feces, FL Medical, Italy and one FecalSwab^TM^, Copan Diagnostics Inc.), and instructed to transfer about 50 g of feces into the Coprocontainer and a swab into the provided Fecal Swab device to deliver for DNA extraction and to store the sample at 4°C. DNA was extracted as soon as the sample was received and stored at -80°C. In order to guarantee the best possible representativeness of the whole gut microbial community, two independent total DNA extractions were conducted from each participant’s fecal sample by column based kit QIAamp DNA Stool Mini (Qiagen, United States).

### Analysis of 16S rRNA Sequences

Amplicons of V3–V4 regions of the 16S rDNA gene were sequenced on an Illumina MiSeq platform (LGC Genomics GmbH, Berlin, Germany^4^) using the universal bacterial primer set S-D-Bact-0341-b-S-17/S-D-Bact-0785-a-A-21 (Herlemann et al., 2011). Data pre-processing included demultiplexing of all samples using Illumina’s CASAVA data analysis software, clipping of Illumina adapters, primer detection and clipping and quality checking of reads during the merging of the paired-end fragments. Subsequently only sequences with a minimal mean quality score of 32 over a moving window of 50 bases were dereplicated resulting in an abundance value for every sequence. Sequences with an abundance value of less than two were discarded. Remaining sequences were pairwise aligned with an altered Smith–Waterman-algorithm (which allows for reduced homopolymer gap costs and semi-global alignment) to calculate the genetic distance. An Expectation–Maximization (EM) algorithm (similar to the PCR error removal in amplicon noise (Quince et al., 2011) with sigma value of 250 was used to cluster the sequences into operational taxonomic units (OTUs). To initialize the EM algorithm, sequences were grouped together by the following procedure: (i) For each sequence the sum of abundances of every other dereplicated sequence within a distance of 3% was calculated. (ii) Sequences were sorted according to the sum from step one in a descending order. (iii) All sequences with a distance less than 3% were grouped to this sequence in an iterative process beginning with the first sequence in the sorted list; this procedure was repeated for all ungrouped sequences until no more sequences were left. (iv) For each group the initial sequence is the representative of the grouped sequences, all other sequences are regrouped to these initial sequences minimizing their distance. For all OTUs representative sequences the closest relative was calculated by the minimal pairwise aligned distance to SILVA 115 non-redundant database resulting in the taxonomy of and the genetic distance to the closest relative with a minimum of 90% similarity (Quast et al., 2013).

The final OTU table comprised 2118 OTUs in a total of 202 samples (101 stool samples with 2 technical replicates each). If consistency within a subject’s DNA extraction replicates (i.e., replicas pertaining to the same subject were required to always be the nearest neighbors in a hierarchical clustering analysis) could be verified; only the replicate showing the highest library size was retained for the analysis. Finally, a subset of OTUs were considered according to several criteria: (i) OTUs assigned to mitochondria or chloroplasts or a domain other than Bacteria (i.e., Archaea and Eukarya) were removed; (ii) OTUs assigned to the Phylum Cyanobacteria were removed due to the interference of chloroplast-derived 16S rDNA; (iii) two OTUs were removed after the calculation of the phylogenetic tree (see below) due conspicuously long branches suggesting chimeric origin; and (iv) OTUs not having complete taxonomic assignation were assigned to the lowest taxonomic level available. When not specified differently, all analyses were conducted on OTU and genera relative abundances.

### Statistical Analysis

#### Microbiota Profiling and Diversity Analysis

Statistical analysis was carried out using R (version 3.2.1) (R Core Team, 2015) software packages and in-house scripts. First, samples were characterized in terms of alpha diversity: sample richness was explored in terms of observed number of species and Chao1 index (Chao, 1987); sample evenness was explored using Simpson and Inverse Simpson indices; overall sample diversity was explored using the Shannon index using the *aindex* function from *DiversitySeq* package (Finotello et al., 2016). The overall difference in the alpha diversity of the samples between V, VG, and O were tested using ANOVA and pairwise comparisons were computed using Tukey’s Honest Significant Differences; both tests were corrected for library size interaction. Then, beta diversity between samples was measured in terms of unique/shared species between the three groups and using both weighted and unweighted UniFrac distance (Lozupone and Knight, 2005; McMurdie and Holmes, 2013). Alpha and beta diversity analysis was performed at all taxonomic levels (OTU, Genus, Family, Order, Class, and Phylum); count tables for higher taxonomic levels were obtained by collapsing OTU abundances based on taxonomical assignation. The maximum likelihood tree (RAxML) used for UniFrac distance computation on the OTU table was calculated using the central/representative sequence of each OTU using ARB (Ludwig et al., 2004) and the CIPRES Science Gateway online platform (Stamatakis et al., 2008). All taxa were considered, even if they were present in one sample only.

Principal component analysis (PCA), Principal Coordinate Analysis (PCoA), and hierarchical clustering based on Bray–Curtis distance were used to investigate possible sample clustering by metadata factors. Two supervised learning methods Random Forest (RF) (Liaw and Wiener, 2002) and Sparse Linear Discriminant Analysis (sLDA) (Knights et al., 2011; Kuhn and Clemmensen, 2012) were tested in an effort to build descriptive models of the data and perform feature ranking on both OTU- and genera-level data.

Differential analysis of OTU and genera in the three groups was carried out on both un-normalized and normalized sequence counts; normalization using TMM (Robinson and Oshlack, 2010) was performed in place of simple proportions or rarefaction in order to take into account data heteroscedasticity (McMurdie and Holmes, 2014). Data normalization was conducted in order to exclude the potential confounding effect of sequencing depth in differential abundance analysis. Non-parametric Kruskal–Wallis testing was carried out, and the false discovery rate (FDR) (Benjamini and Hochberg, 1995; Storey and Tibshirani, 2003) values were estimated using the Benjamini–Hochberg method to control for multiple testing.

Prevalent taxa for each sample were investigated and associated to each sample’s enterotype (obtained according to the original manuscript (Arumugam et al., 2011; Wu et al., 2011); moreover, possible enterotype-diet associations were tested using Pearson’s Chi-Squared test and quantified by Cramer’s V measure. Possible correlations between microbiota composition and anthropometric data were investigated using Spearman’s correlation coefficient.

#### Dietary Profiles

Micro- and macro-nutrient data were explored through PCA and Random Forest to investigate possible group-related clustering and to perform feature ranking. Differences in samples’ micro-nutrient abundance between V, VG, and O were tested using the Kruskal–Wallis non-parametric test. Possible correlation between micro-nutrient abundance, anthropometric data, and microbiota composition at genus level were investigated using Spearman’s correlation coefficient. Nutritional data were converted to average caloric percentage of lipids, proteins, and carbohydrates (using the following conversion: 1 g fat = 9 kcal, 1 g protein = 4 kcal, 1 g carbohydrates = 4 kcal) and were then compared to standard guidelines for a healthy diet (Oksanen et al., 2016). They were also tested for differential abundance between V, VG, and O using the Kruskal–Wallis test and correlations with anthropometric data and microbiota alpha diversity were computed. A Mantel-test was conducted to test for a correlation between variations in nutrient intake (Euclidean distance of relative abundance of dietary composition) to the beta-diversity (Bray–Curtis distance of relative genera abundances profiles of each participant).

#### Analysis of Variance Using Beta Diversity Distance Matrix

Permutational Multivariate Analysis of Variance Using Distance Matrices (PERMANOVA using the *Adonis* command in the *Vegan* package) (NIH, 2016) with the Bray–Curtis distance computed on proportional genera profiles was used to investigate the influence of all analyzed factors in contributing to the observed beta diversity among the microbiota profiles. All available continuous and categorical variables were considered: group, enterotype, richness (expressed as observed number of species), evenness (expressed as normalized Shannon index, i.e., Shannon index divided by its maximum value), mean lipid, protein and carbohydrate consumption, BMI, BFM, gender, and province of origin of the participant. Firstly, each variable was tested for significance in explaining the variance of the measured beta-diversity; then, pairs of variables and their interaction were tested for significance in partitioning the measured beta-diversity. All variables and interactions with a *p*-value <0.2 (for a conservative approach to feature selection), were tested in a multivariable complex model. Finally, only the variables proven to be significant in the complete model were retained. Indeed, a parsimony approach was used, trying to select for the simplest model possible to partition data variability with significant variables. This approach, therefore, could possibly explain a smaller amount of the total variability compared to the complete model, but avoided considering variables that explain a small amount of variability due to spurious significance. A reduced model was built in which, by discarding non-significant variables (or their interaction), more degrees of freedom were available to stabilize the pseudo-*F* test. Each model was built using 9999 random permutations.

## Results

### Sample Description

The three groups (O, VG, and V) of participants of the study were not significantly different in terms of age, gender, BMI, BLM, or BFM (**Table 1** and Supplementary Figure S1). Anthropometric data for each sample are detailed in Supplementary Results 1.

**Table 1:** Characteristics of participants in the study belonging to the cohorts Omnivorous (O), Vegetarian (V), and Vegan (VG): Sample size (N), Females %, average value ± standard deviation of Age, body mass index (BMI), body lean mass (BLM), body fat mass (BFM).

Diet	*N*	Females %	Age	BMI	BLM %	BFM %
*O*	43	73.3	45.0 ± 13.9	23.8 ± 4.7	76.1 ± 9.1	24.9 ± 9.1
*V*	32	70	42.3 ± 13.2	23.8 ± 9.1	76.1 ± 8.9	24.9 ± 8.9
*VG*	26	65.3	39.4 ± 11.1	23.7 ± 3.4	75.4 ± 7.2	25.6 ± 7.2
Total	101	68.3	42.5 ± 13.0	23.8 ± 4.4	75.0 ± 8.7	24.9 ± 8.7

*Percentage of male participants can always be computed as 100%-Females %*.

### Dietary Profiles

**FIGURE 1:**
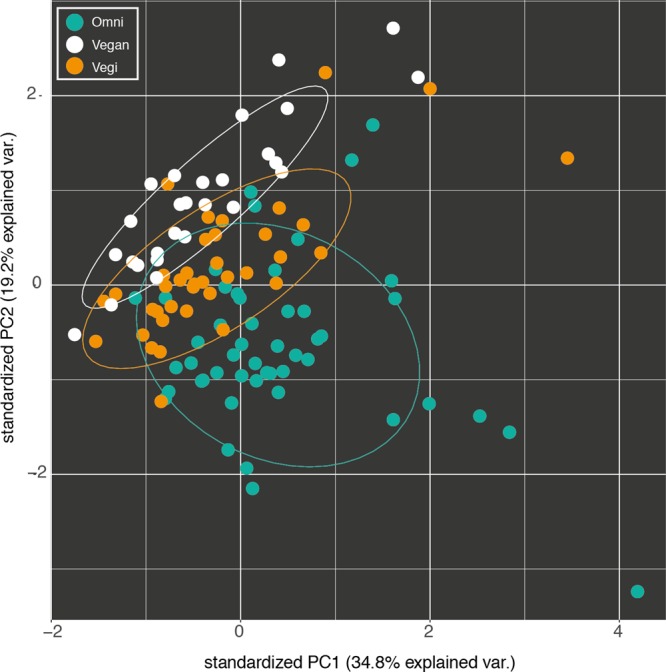
Fist two components of the principal component analysis (PCA) of the nutrient intake profiles of the omnivorous (Omni), vegan and vegetarian (Vegi) participants in the study. The variance explained by each component is written in brackets.

The dietary profiles of the three groups can be distinguished starting from their average nutritional intake (**Figure 1**). Indeed, 57 out of 76 nutrients proved to be differentially distributed among the three groups (Kruskal–Wallis test with 5% level of significance). Consequently, supervised learning techniques discriminated and predicted the three classes (O, VG, and V) with an acceptable error (Random Forest Out-of-bag estimate of classification error rate: OOB = 10.43%). Moreover, the available variables were ranked by their importance in discriminating between the three groups, using the mean decrease in accuracy (Supplementary Figure S2): average consumption of *animal proteins* and *cholesterol*, and the two Omega-3 fatty acids, *EPA* and *DHA*, were the top-ranking ones.

**FIGURE 2:**
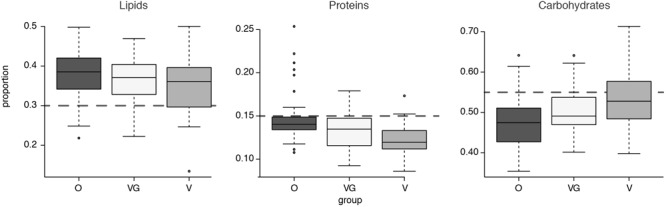
Boxplot of average caloric intake for each macrocategory of lipids, carbohydrates, and proteins, divided into the three groups (O = Omnivorous, V = Vegetarian, and VG = Vegan). The gray dashed lines highlight expected intakes according to the guidelines.

Nutritional profiles were further summarized in terms of the total amount and percentage of total calories (Kcal) imputed to each of the three macro-nutrient categories of lipids, proteins, and carbohydrates (**Figure 2**). When compared with the standard guidelines for healthy average intakes of these components (30% of total energy intake from fats, 15% from proteins and 55% from carbohydrates) (NIH, 2016), the groups proved to be higher than suggested for average calorie intake from lipids and lower than suggested from proteins (**Figure 2**). Nutrient data for each sample are detailed in Supplementary Results 1. When the differential abundance between the groups was examined (Kruskal–Wallis test with 5% level of significance), lipids showed no significant differential distribution among the three groups (*p*-value = 0.13); proteins showed significant differential distributions between O and plant-based diets [both VG (*p*-value < 0.0001) and V (*p*-value = 0.0223)]; carbohydrates showed a significant differential distribution between Omnivores and Vegetarians only (*p*-value = 0.0004).

### Sequencing Depth and Reproducibility

A total of 4.8 × 106 reads were produced by the sequencing runs. The microbial community of the two DNA extractions for each participant always clustered together (hierarchical clustering computed on Bray–Curtis distance of microbial profile, data not shown), indicating that the bacterial community profiling was consistently reproducible within individual. For this reason, subsequent analyses were conducted using the replicate with the higher sequencing depth. A total of 1130000 reads were used for analysis after normalization. The resulting OTU table comprised 1872 OTUs with 253 singletons and 101 samples, with a mean library size of 11080 ± 6704.399, range [1535–29840]. Combined sequences of the replicates used in the study were deposited in the European Nucleotide Archive (ENA) with study accession PRJEB18693.

### Microbiota Profiling and Diversity Analysis

Microbiota profiles were investigated in terms of taxonomic assignment, most abundant taxa and relative abundance. A total of 1872 OTUs were found, accounting for 212 genera, 102 families, 61 orders, 39 classes, and 20 phyla. The two phyla that dominated the relative read abundances were Bacteroidetes [median = 49.1% of all reads, interquartile range IQR = (34.96–64.17%)] and Firmicutes [median = 40.61% of all reads, IQR = (31.43–52.99%)] followed by Proteobacteria and Verrucomicrobia [with median = 3.02% and IQR = (1.59–5.79%) and median = 0.11% and IQR = (0.01–0.96%), respectively]. Supplementary Figure S3 shows the stacked bar plots of proportional abundances of phyla for each recruited sample.

Each taxon was tested for differential abundance (both at OTU and genus level) between the three sampled groups (O, V, and VG). When correcting for multiple testing using an overall FDR rate of 5% to check for false positives, only four OTUs from three genera were found to be differentially abundant in the three groups. These OTUs were affiliated with *Bacteroides, Lachnospiraceae*, and *Ruminococcaceae*. However, each of the highlighted OTU accounted for less than 1% of counts in the individual samples (data not shown).

**FIGURE 3:**
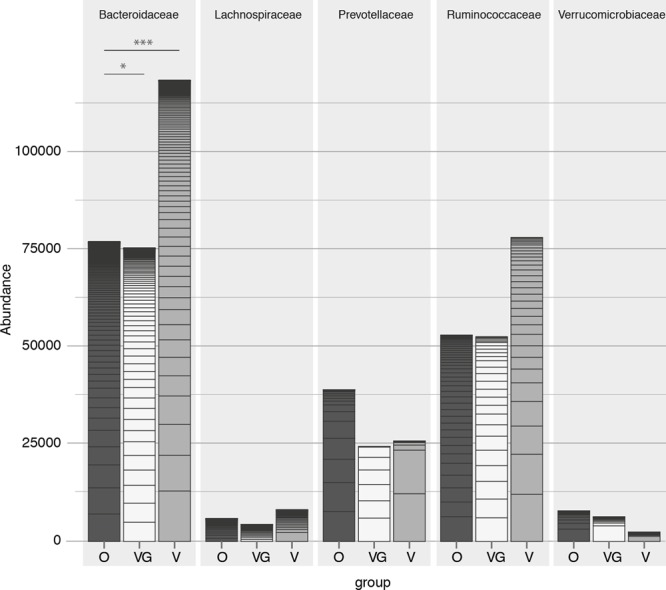
Count abundances of the five most abundant *families*, with different contributions toward each of the three groups (O = Omnivorous, V = Vegetarian, and VG = Vegan). *p*-value: ^∗^ < 0.05, ^∗∗^ < 0.01, ^∗∗∗^ < 0.001.

On a higher taxonomic level, the number of counts assigned to Bacteroidetes was significantly different among groups (*p* = 0.002) (Kruskal–Wallis test with 5% level of significance, **Figure 3**), with higher counts in VG and V compared to O (VG–O *p* = 0.013, V–O *p* = 0.006). Conversely, no significant difference was observed in the Firmicutes/Bacteroides ratio distribution among the three groups, or in the Prevotellaceae abundance distribution (**Figure 3**). The same data were used to characterize each enterotype with its prevalent family (*Bacteroidaceae, Prevotellaceae*, or *Ruminococcaceae*, respectively). The proportion of samples assigned to each of the three enterotypes was independent from the feeding type group (Pearson’s chi-squared test with 5% level of significance and Cramer’s V measure of association; **Table 2**). Genera proportional abundance for each sample is detailed in Supplementary Results 1. Additionally, correlation of anthropometric data, diet and microbiota were investigated as detailed in Supplementary Results 2.

**Table 2:** Distribution of enterotypes between the three groups (O, VG, and V), Person chi square significance and Cramer’s *V*-value

Enterotype	Omnivorous (O)	Vegan (VG)	Vegetarian (V)
Bacteroidaceae	21	11	11
Prevotellaceae	9	5	5
Ruminococcaceae	13	11	16
*P*-value Pearson Chi^2^: 0.5487		Cramer’s V: 0.122375	

**FIGURE 4:**
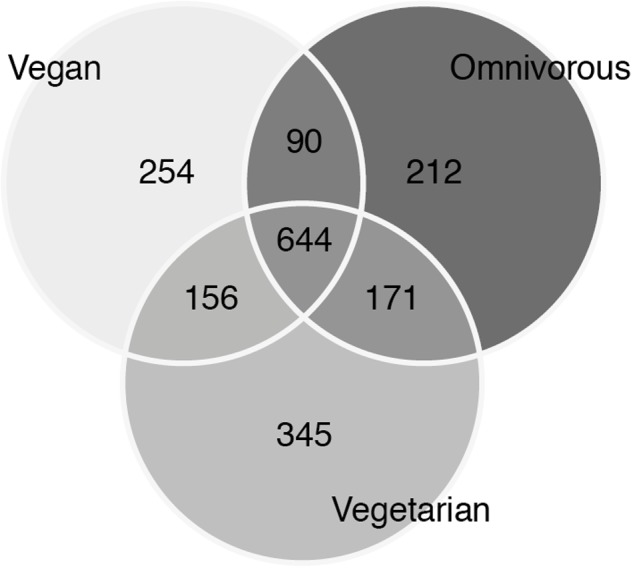
Venn diagram of unique and shared operational taxonomic units (OTUs) between the three groups.

Alpha diversity was lower in O compared to V both in terms of actual OTU richness and estimates by the Chao1 indices (*p*-value <0.05; **Tables 3A,B**). The differences were statistically significant even when sequencing depth was considered as a confounding factor. The overall alpha diversity of the samples was also tested using iSimpson, cSimpson, and Shannon indices, but they presented no difference between the three groups (Supplementary Table S1). The beta diversity of the samples was characterized in terms of shared and unique species between the three groups (**Figure 4**). All the OTUs that were found in more than 90% of the samples within each group, were also shared between all three groups.

**Table 3A:** Richness of the microbial community of Omnivorous (O), Vegans (VG), and Vegetarians (V): ANOVA summary results and Tukey’s pairwise comparisons on sample alpha diversity.

		*N*	ALPHA: Richness	*Df*	Sum of squares	*F*-value	*p*-value
Group	O	43	146.7907	2	28039	4.4362	0.01429^∗^
	VG	26	175.4444				
	V	32	183.4688				
Residuals				99		3	
Overall		101	165.8824		312866		

**Group comparisons**		**Difference**	**Lower**	**Upper**	**Adjusted *p*-value**	**Significance**	

VG vs. O		28.653747	–4.191709	61.49920	0.1001182	.	
V vs. O		36.678052	5.448641	67.90746	0.0170229	^∗^	
V vs. VG		8.024306	–26.930872	42.97948	0.8486384	

*Significance levels: p-value: ^.^ < 0.10; ^∗^ < 0.05, ^∗∗^ < 0.01. N = number of observations; df = degree of freedom*

**Table 3B:** Chao1 of the microbial community of Omnivorous (O), Vegans (VG), and Vegetarians (V): ANOVA summary results and Tukey pairwise comparisons on sample alpha diversity.

		*N*	ALPHA: Chao	*Df*	Sum of squares	*F*-value	*p*-value
Group	O	43	207.9632	2	46000	4.0388	0.0206^∗^
	VG	26	246.3368				
	V	32	254.1096				
Residuals				99		8	
Overall		101	232.5982		563784		

**Group comparison**		**Difference**	**Lower**	**Upper**	**Adjusted *p*-value**	**Significance**	

VG vs. O		38.373588	–5.717685	82.46486	0.1011775	.	
V vs. O		46.146384	4.224466	88.06830	0.0273073	^∗^	
V vs. VG		7.772796	–39.150538	54.69613	0.9180056	

*Significance levels: p-value: ^.^ < 0.10; ^∗^ < 0.05, ^∗∗^ < 0.01. N = number of observations; df = degree of freedom*

**FIGURE 5:**
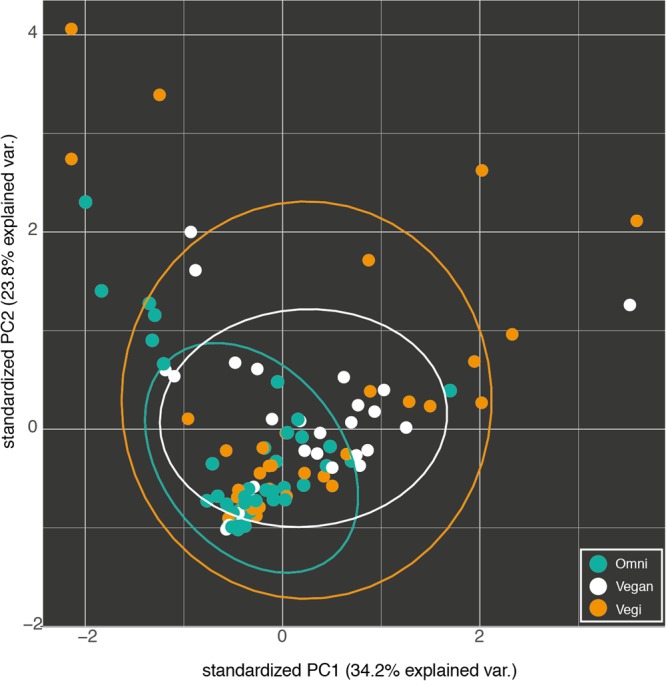
Fist two components of the PCA of the OTU profiles of the omnivorous (Omni), vegan and vegetarian (Vegi) participants in the study. The variance explained by each component is written in brackets.

Therefore, a vast proportion of the microbiota in our samples can be referred to as a shared ‘core’ microbiome. OTUs that were unique for each group could be mostly attributed to the individual contribution of one or a few samples. Those included both OTUs found in one sample only (677 of 1872) as well as 253 singletons (OTUs with one count only). Several beta diversity measures were tested, including Bray–Curtis, Canberra and phylogenetic-based diversity (unweighted and weighted UniFrac distance), and were used both for the hierarchical clustering of the samples and for dimensionality reduction analysis such as PCA/PCoA. Since all the analyses gave similar results, only the results of the PCA are shown (**Figure 5**). None of the conducted analyses resulted in a differential clustering of the microbial communities by the hosts’ feeding group (O–V–VG). Similar patterns of data overlap were found when different labels (gender, enterotype, BMI category) were investigated instead of the feeding group (data not shown). Consequently, both supervised learning techniques employed (RF and sLDA) were unable to infer group based on microbiota profiling as reported in Supplementary Table S2 (out-of-bag estimate of error rate for RF: OOB = 50.98 % at OTU level).

Moreover, Mantel test highlighted that Euclidean distances of total detailed nutritional intake pattern did not significantly correlate with the distances of the microbial community composition (Bray–Curtis on genera proportion) between participants (*r* = 0.056, *p* = 0.19).

### Analysis of Variance Using Beta Diversity Distance Matrix

In order to discover variables that influenced total bacterial community composition, PERMANOVA analysis on Bray–Curtis distance was computed on the relative abundances of genera. After testing each variable individually and its interaction with all other variables (data not shown), the variables achieving *p*-value <0.2 were tested in a complex model (Supplementary Table S3).

Most of the factors, although achieving significance, explained less than 2% of total measured variance, while 44.16% of total variance remained unexplained by the selected variables. The reduced model, built using significant variables only, highlighted that sample enterotype, richness (expressed as observed number of OTUs), evenness and body fatty mass were the most significant individual variables in partitioning the measured distance (*p* < 0.05) (**Table 4**). Most variance was explained by sample enterotype (36.75% of total variance) followed by richness and evenness (1.657 and 2.911%, respectively) while body fatty mass was the least influential of the significant factors (1.14% of total variance).

**Table 4:** Results of the analysis of variance of the Beta-diversity (ADONIS) using parsimoniously reduced statistical model containing only significant variable.

	*Df*	Sums of squares	Mean of squares	*F*-value	*R*^2^	*p*-value	Significance
Enterotype	2	6.0526	3.02628	33.706	0.36750	0.0001	^∗∗∗^
Richness	1	0.2729	0.27295	3.040	0.01657	0.0091	^∗∗^
Normalized shannon	1	0.4794	0.47936	5.339	0.02911	0.0002	^∗∗∗^
BFM	1	0.1878	0.18775	2.091	0.01140	0.0482	^∗^
Region	6	0.7059	0.11765	1.310	0.04286	0.1000	.
Group	2	0.2583	0.12915	1.438	0.01568	0.1204	
Proteins	1	0.1679	0.16786	1.870	0.01019	0.0687	.
Richness:Group	2	0.3255	0.16274	1.813	0.01976	0.0389	^∗^
Enterotype:Normalized shannon	2	0.5847	0.29234	3.256	0.03550	0.0007	^∗∗∗^
Richness:Normalized shannon	1	0.1077	0.10768	1.191	0.00654	0.2762	
Richness:Proteins	1	0.1618	0.16184	1.803	0.00983	0.0772	.
Residuals	81	7.2727	0.08979		0.44159		
Total	100	16.4694	1.00000				

*Significance levels: p-value: ^.^ < 0.10; ^∗^ < 0.05, ^∗∗^ < 0.01. ^∗∗∗^ < 0.001. Df = degree of freedom*

The interactions between variables that were significant in partitioning the measured data variability were: richness by host group (O, V, and VG) (1.97% of total variance), highlighting that sample distance varies with richness differently among the groups; evenness by enterotype (3.55% of total variance), highlighting that sample distance varies with evenness differently among the enterotypes.

## Discussion

The existence of a functional connection between diet and gut microbiota is well established (De Filippo et al., 2010; De Filippis et al., 2016). Thus, recent trends of nutritional therapy apply the beneficial use of diet to improve human health, through gut microbiota performances (Cho and Blaser, 2012). However, even though diet composition is known to have a modulating influence on the gut microbial communities, knowledge on the role exerted by specific nutrients in driving gut microbial assortment is still limited.

In the last few years, a plethora of research has focused on vegan and vegetarian diets as experimental dietary conditions aimed at mitigating inflammatory diseases and metabolic syndrome (Peltonen et al., 1997; Kjeldsen-Kragh, 1999; Tonstad et al., 2013; Glick-Bauer and Yeh, 2014; Jenkins et al., 2014; Le and Sabaté, 2014; Tuso et al., 2015). However, standardization for prognosis purposes is hard to address due to the variety of foodstuffs that can be included in such dietary styles. Moreover, few studies rigorously evaluate and compare omnivorous, vegetarian, and vegan subjects as distinct experimental groups (Power et al., 2014). Therefore, it is difficult to discern whether the microbiota-derived features that are attributed to fiber-rich diets (compared with fat and protein of animal origin diets), and the derived advantages for health, could be generalized to the whole vegan/vegetarian dietary style, independently of food content in terms of nutrients and associated calories.

Here the specific impact of vegan, vegetarian, and omnivore food choices on the composition of gut microbiota was investigated among relatively homogeneous groups for variables known to have a role in modulating gut microbial composition such as age, anthropometric variables, ethnicity and geographic area. Interestingly here we found that there were broad differences between the cohorts, which were, however, not reflected when analyzing the total bacterial composition.

Statistically significantly different alpha diversity indices were found between the gut microbiota of vegetarians and omnivores, with vegetarians displaying greater richness. The effects of the richness of the gut microbiota on the host’s health are still debated. It is intriguing to speculate that a richer gut microbiota is advantageous to the host, since greater taxonomic richness might also mean greater functional diversity, there is, however still little empirical evidence to support this notion (Cho and Blaser, 2012; Clemente et al., 2012; Marchesi et al., 2016).

The Bacteroidetes phylum was prevalent among all the three investigated groups, and a statistically significant difference in their relative counts between vegans or vegetarians and omnivores was observed. In other studies this family has been found to be adapted to the gut conditions of people with a typical *westernized* diet (Power et al., 2014). Here we show, however, that within an example of a Western society, the contribution of this genotype differ, and that their abundance might be specifically related to a low intake of animal protein.

On the contrary to total richness and Bacteroidetes abundances, the three dietary types could not be distinguished when analyzing the whole community composition, i.e., beta-diversity. The three addressed groups displayed a shared *core microbiota*, probably due to their common intake in terms of nutrients rather than food. One common factor between the groups was high fat content and reduced protein and carbohydrate contributions to diet, which might mitigate the differences between the compositions of microbiota and drive microbial communities toward a *westernized* profile. Difference in composition was rather found in the rare microbiota, which, however, was not enough to explain differences between the dietary groups. This was in accordance with previous work by De Filippis et al., (2016), where no difference in microbial composition of groups differently adhering to the Mediterranean Diet was found.

Among the whole dataset, the three described enterotype classes could be clearly distinguished, although no clear link between each investigated dietary group and enterotype could be seen. This might be because of the displayed high inter-individual variability in microbial composition observed in our sample, which confirmed a well-known trend in human gut microbiota studies (De Filippis et al., 2016). Moreover, this enterotype-based signature explained approximately 37% of the total variance in beta diversity, with other diversity related parameters (richness and evenness) accounting for approximately 5% of the total variance, while other factors and their interactions explained an additional small amount (10.4%) of total variance. However, 44% of the total variance could not be explained by any of the investigated variables. Other variables known to contribute in shaping gut microbiota could come into play in this context, including participants’ extent of physical activity (Clark and Mach, 2016), diet history (Jeffery et al., 2012), and their dietary literacy.

Here we show, that the gut microbiome of vegan, vegetarians, and omnivores differ in terms of total richness and abundance of Bacteroidetes. This study, however, also demonstrates that the use of the labels vegan/vegetarian/omnivorous to infer conclusions on the detailed gut microbial composition appear to be of limited discriminatory potential. This might be because these labels do not sufficiently define the dietary composition in terms of nutrients that shapes the gut microbiota. Thus, the situation where the categorization of individuals on the basis of their claimed feeding types is used for planning studies on the impact of food consumption on gut microbial composition appear to be likely oversimplified.

## Ethics Statement

All subjects involved gave their written informed consent for the inclusion in the study. The protocol was approved by the Ethics Committee of the Istituto Zooprofilattico Sperimentale delle Venezie.

## Author Contributions

CL planned and executed the laboratory work, evaluated the data, and wrote the paper. CL and VC recruited the participants and provided the metadata. EE, EM, and MM performed the bioinformatics and biostatistics and wrote the paper. EE, JV, and JP provided and pre-processed the sequence data on an in-house pipeline programmed by JV. ADC performed the laboratory work and provided input for writing the paper. FB provided critical input for execution of pre-processing. IP provided critical input for execution of biostatistics tests. CL, GC, and AR initiated and planned the study, evaluated the experiments, and wrote the paper. All authors read and approved the final manuscript.

## Conflict of Interest Statement

The authors declare that the research was conducted in the absence of any commercial or financial relationships that could be construed as a potential conflict of interest.

## Footnotes

1 http://www.scienzavegetariana.it/

2 http://www.lav.it

3 https://dietassessmentprimer.cancer.gov/approach/table.html

4 www.lgcgroup.com/genomics
