# Supplementary material for: Assessing the Influence of Vegan, Vegetarian and Omnivore Oriented Westernized Dietary Styles on Human Gut Microbiota: A Cross Sectional Study
Source: Front Microbiol. 2018 Mar 5;9:317. doi: 10.3389/fmicb.2018.00317 (PMC5844980; doi:10.3389/fmicb.2018.00317)
Supplement: Supplementary file 1 [file Table_1.docx]

Supplementary Table 1: Alpha diversity summary: minimum value (min), 1° quantile (1st Qu), median value (median), mean vale (mean), 3° quantile (3st Qu) and maximum value (Max)

|  | **Richness** | **Chao** | **iSimpson** | **cSimpson** | **Shannon** |
| --- | --- | --- | --- | --- | --- |
| **Min.** | 62 | 79 | 1.888 | 0.4705 | 1.373 |
| **1st Qu.** | 115.2 | 173.6 | 4.104 | 0.7563 | 2.314 |
| **Median** | 165.5 | 235 | 7.154 | 0.8602 | 2.754 |
| **Mean** | 165.9 | 232.6 | 8.565 | 0.8337 | 2.749 |
| **3rd Qu.** | 200 | 282.7 | 11.36 | 0.912 | 3.266 |
| **Max.** | 340 | 462.6 | 30.68 | 0.9674 | 4.07 |
