# Supplementary material for: Assessing the Influence of Vegan, Vegetarian and Omnivore Oriented Westernized Dietary Styles on Human Gut Microbiota: A Cross Sectional Study
Source: Front Microbiol. 2018 Mar 5;9:317. doi: 10.3389/fmicb.2018.00317 (PMC5844980; doi:10.3389/fmicb.2018.00317)
Supplement: Supplementary file 2 [file Table_2.docx]

Supplementary Table 2: Random Forest (RF) and Sparse Linear Discriminant Analysis (sLDA) performance in clustering microbiota profiles into groups. OOB=out of bag error rate.

|  | **OTU** | | | **Genera** | | |
| --- | --- | --- | --- | --- | --- | --- |
| **RF** | Class | Class Error | OOB error rate | Class | Class Error | OOB error rate |
|  | Omni (0) | 0.1162791 | 50.98% | Omni (0) | 0.2093023 | 54.9% |
|  | Vegi (V) | 0.9062500 |  | Vegi (V) | 0.6875000 |  |
|  | Vegan (VG) | 0.9629630 |  | Vegan (VG) | 0.9259259 |  |
| **sLDA** | Class | Class Error | Accuracy | Class | Class Error | Accuracy |
|  | Omni (O) | 0.88372093 | 50.00% | Omni (O) | 0.81395349 | 52.94% |
|  | Vegi (V) | 0.09375 |  | Vegi (V) | 0.03125 |  |
|  | Vegan ()VG | 0.37037037 |  | Vegan (VG) | 0.7037037 |  |
