# Supplementary material for: Assessing the Influence of Vegan, Vegetarian and Omnivore Oriented Westernized Dietary Styles on Human Gut Microbiota: A Cross Sectional Study
Source: Front Microbiol. 2018 Mar 5;9:317. doi: 10.3389/fmicb.2018.00317 (PMC5844980; doi:10.3389/fmicb.2018.00317)
Supplement: Supplementary file 3 [file Table_3.docx]

Supplementary Table 3: Results of the analysis of variance of the Beta-diversity (ADONIS) using the complete model. Significance levels: p-value: 0 ‘***’ 0.001 ‘**’ 0.01 ‘*’ 0.05 ‘.’ 0.1 ‘ ’ 1. Df=degree of freedom; Signif.=significance level.

|  | **Df** | **Sums of Squares** | **Mean of Squares** | **F value** | **R2** | **p-value** | **Signif.** |
| --- | --- | --- | --- | --- | --- | --- | --- |
| **enterotype** | 2 | 5.1282 | 2.56411 | 24.8797 | 0.27099 | 0.0001 | *** |
| **richness** | 1 | 1.0545 | 1.05446 | 10.2315 | 0.05572 | 0.0001 | *** |
| **normalized shannon** | 1 | 0.9502 | 0.95018 | 9.2196 | 0.05021 | 0.0001 | *** |
| **lipids** | 1 | 0.1658 | 0.16583 | 1.609 | 0.00876 | 0.1027 |  |
| **proteins** | 1 | 0.1791 | 0.17912 | 1.738 | 0.00947 | 0.0767 | . |
| **carbohydrate** | 1 | 0.1697 | 0.16967 | 1.6463 | 0.00897 | 0.0927 | . |
| **bmi** | 1 | 0.1527 | 0.1527 | 1.4816 | 0.00807 | 0.139 |  |
| **sex** | 1 | 0.1558 | 0.15577 | 1.5114 | 0.00823 | 0.1284 |  |
| **region** | 6 | 0.8142 | 0.1357 | 1.3167 | 0.04303 | 0.0828 | . |
| **group** | 2 | 0.3424 | 0.1712 | 1.6612 | 0.01809 | 0.0482 | * |
| **bfm** | 1 | 0.2689 | 0.26887 | 2.6089 | 0.01421 | 0.0079 | ** |
| **richness:group** | 2 | 0.2453 | 0.12263 | 1.1899 | 0.01296 | 0.2496 |  |
| **carbohydrate:group** | 2 | 0.3111 | 0.15553 | 1.5091 | 0.01644 | 0.0785 | . |
| **sex:group** | 2 | 0.2437 | 0.12183 | 1.1821 | 0.01288 | 0.2562 |  |
| **enterotype:richness** | 2 | 0.3873 | 0.19364 | 1.8789 | 0.02047 | 0.0196 | * |
| **enterotype: normalized shannon** | 2 | 0.5361 | 0.26805 | 2.6009 | 0.02833 | 0.0011 | ** |
| **enterotype:proteins** | 2 | 0.1638 | 0.08188 | 0.7945 | 0.00865 | 0.7131 |  |
| **enterotype: carbohydrate** | 2 | 0.1393 | 0.06967 | 0.676 | 0.00736 | 0.8486 |  |
| **richness: norm shannon** | 1 | 0.2349 | 0.23485 | 2.2788 | 0.01241 | 0.022 | * |
| **richness:lipids** | 1 | 0.1904 | 0.19044 | 1.8478 | 0.01006 | 0.0591 | . |
| **richness:proteins** | 1 | 0.1472 | 0.14723 | 1.4285 | 0.00778 | 0.1576 |  |
| **richness: carbohydrate** | 1 | 0.0821 | 0.0821 | 0.7966 | 0.00434 | 0.6103 |  |
| **richness:region** | 5 | 0.4457 | 0.08913 | 0.8649 | 0.02355 | 0.7161 |  |
| **normalized shannon:lipids** | 1 | 0.2127 | 0.21275 | 2.0643 | 0.01124 | 0.037 | * |
| **proteins:bfm** | 1 | 0.1359 | 0.13586 | 1.3183 | 0.00718 | 0.2059 |  |
| **carbohydrate:bfm** | 1 | 0.0938 | 0.09383 | 0.9105 | 0.00496 | 0.4907 |  |
| **sex:bfm** | 1 | 0.1246 | 0.1246 | 1.209 | 0.00658 | 0.2599 |  |
| **sex:bmi** | 1 | 0.1765 | 0.17655 | 1.7131 | 0.00933 | 0.0797 | . |
| **sex:region** | 4 | 0.5191 | 0.12978 | 1.2593 | 0.02743 | 0.1493 |  |
| **Residuals** | 50 | 5.153 | 0.10306 | 0.2723 |  |  |  |
| **Total** | 100 | 18.9239 | 1 |  |  |  |  |
