# Supplementary material for: Assessing the Influence of Vegan, Vegetarian and Omnivore Oriented Westernized Dietary Styles on Human Gut Microbiota: A Cross Sectional Study
Source: Front Microbiol. 2018 Mar 5;9:317. doi: 10.3389/fmicb.2018.00317 (PMC5844980; doi:10.3389/fmicb.2018.00317)
Supplement: Supplementary file 4 [file Table_4.docx]

Supplementary Table 4: Spearman’s correlation coefficients (rho) between micro- and macro-nutrients and anthropometric data. Correlations significantly different from zero are highlighted in bold.

| **COMPONENT** | **BMI** | **BFM** | **BLM** |
| --- | --- | --- | --- |
| **Energy kcal** | -0.032583498 | **-0.312631226** | **0.312631226** |
| **Alchool g** | 0.027302331 | -0.174434027 | 0.174434027 |
| **Proteins g** | 0.088413463 | **-0.223090028** | **0.223090028** |
| **Lipids g** | -0.077841591 | **-0.333982731** | **0.333982731** |
| **Carbohydrates available g** | -0.046988036 | **-0.257860481** | **0.257860481** |
| **Starch g** | 0.079117205 | -0.137678184 | 0.137678184 |
| **Total fibers g** | -0.183577778 | **-0.251517931** | **0.251517931** |
| **Soluble fibers g** | -0.07881432 | -0.164585972 | 0.164585972 |
| **Insoluble fibers g** | -0.179698513 | -0.138988628 | 0.138988628 |
| **Cholesterol mg** | 0.066406727 | 0.014843785 | -0.014843785 |
| **Animal Proteins g** | 0.142820959 | 0.045025966 | -0.045025966 |
| **Vegetable Proteins g** | -0.081260703 | -0.194079704 | 0.194079704 |
| **Saturate fat g** | -0.036666628 | **-0.227149493** | **0.227149493** |
| **C4:0-C10:0 g** | 0.00431834 | -0.00107291 | 0.00107291 |
| **C12:0 lauric acid g** | -0.002847161 | -0.058163186 | 0.058163186 |
| **C14:0 miristic acid g** | -0.009872933 | -0.04858559 | 0.04858559 |
| **C16:0 palmitic acid g** | 0.105824483 | -0.129286646 | 0.129286646 |
| **C18:0 stearic acid g** | 0.048618959 | **-0.21593209** | **0.21593209** |
| **C20:0 arachid acid g** | 0.037288373 | -0.127598418 | 0.127598418 |
| **C22:0 beenic acid g** | **-0.19866213** | **-0.335010384** | **0.335010384** |
| **Monosaturated fat g** | -0.042433103 | **-0.220061445** | **0.220061445** |
| **C14:1 miristoleic acid g** | 0.088668364 | 0.077910779 | -0.077910779 |
| **C16:1 palmitoleic acido g** | 0.126639679 | -0.065534624 | 0.065534624 |
| **C18:1 oleic acid g** | 0.040126513 | -0.160340133 | 0.160340133 |
| **C20:1 eicosaenoic acid g** | 0.138570867 | -0.089942514 | 0.089942514 |
| **C22:1 erucic acid g** | -0.09634849 | -0.065349711 | 0.065349711 |
| **Polisaturated fat g** | -0.168852426 | **-0.286793442** | **0.286793442** |
| **C18:2 linoleic acid g** | -0.128994304 | **-0.311524629** | **0.311524629** |
| **C18:3 linolenic acid g** | -0.118143878 | **-0.251243477** | **0.251243477** |
| **C20:4 arachidonic acid g** | 0.104368013 | -0.091537667 | 0.091537667 |
| **C20:5 EPA g** | 0.096726847 | 0.016938532 | -0.016938532 |
| **C22:6 DHA g** | 0.068525198 | -0.003309767 | 0.003309767 |
| **Calcium mg** | -0.089525985 | -0.066366721 | 0.066366721 |
| **Sodium mg** | -0.002801692 | **-0.208849867** | **0.208849867** |
| **Potassium mg** | -0.045397887 | -0.180940317 | 0.180940317 |
| **Phosphorous mg** | -0.104681912 | **-0.269875799** | **0.269875799** |
| **Iron mg** | -0.118876761 | **-0.323755442** | **0.323755442** |
| **Magnesium mg** | -0.013169698 | **-0.215518572** | **0.215518572** |
| **Zinc mg** | -0.160727392 | **-0.287482345** | **0.287482345** |
| **Copper mg** | -0.126820986 | **-0.22364141** | **0.22364141** |
| **Selenium mcg** | 0.173780595 | 0.070600911 | -0.070600911 |
| **Thiamine mg** | -0.06589819 | **-0.306173084** | **0.306173084** |
| **Riboflavin mg** | 0.089759235 | -0.089226952 | 0.089226952 |
| **Niacin mg** | 0.078389115 | -0.184126152 | 0.184126152 |
| **Vitamin A mcg** | **-0.220722034** | **-0.196368613** | **0.196368613** |
| **Vitamin C mg** | -0.020374879 | -0.055434703 | 0.055434703 |
| **Vitamin E mg** | -0.046679326 | -0.173508642 | 0.173508642 |
| **Fitic acid g** | -0.172543949 | **-0.261495048** | **0.261495048** |
| **Water g** | 0.019448748 | -0.100065522 | 0.100065522 |
| **Lisin mg** | 0.107961231 | -0.058474934 | 0.058474934 |
| **Histidine mg** | 0.127281836 | -0.096559356 | 0.096559356 |
| **Arginin mg** | 0.073939027 | -0.176100409 | 0.176100409 |
| **Aspartic acid mg** | 0.085600122 | -0.139052694 | 0.139052694 |
| **Threonin mg** | 0.098763994 | -0.116652834 | 0.116652834 |
| **Serin mg** | 0.088326092 | -0.145267112 | 0.145267112 |
| **Glutamic acid mg** | 0.117513776 | -0.190561889 | 0.190561889 |
| **Prolin mg** | 0.107303038 | -0.122348899 | 0.122348899 |
| **Glycin mg** | 0.149409956 | -0.138720715 | 0.138720715 |
| **Alanin mg** | 0.142752299 | -0.091463184 | 0.091463184 |
| **Cystine mg** | 0.143311472 | -0.162704757 | 0.162704757 |
| **Valin mg** | 0.095735139 | -0.123927256 | 0.123927256 |
| **Methionine mg** | 0.120833868 | -0.070781462 | 0.070781462 |
| **Isoleucine mg** | 0.105345931 | -0.114858982 | 0.114858982 |
| **Leucine mg** | 0.098321315 | -0.122197469 | 0.122197469 |
| **Tyrosine mg** | 0.037214152 | -0.123985498 | 0.123985498 |
| **Phenylalanine mg** | 0.07063641 | -0.173077651 | 0.173077651 |
| **Tryptophan mg** | 0.071952797 | -0.137817965 | 0.137817965 |
| **Folic acid mcg** | -0.080427768 | **-0.237761179** | **0.237761179** |
| **Vitamin D mcg** | **0.288078729** | 0.130142811 | -0.130142811 |
| **b-Carotene mcg** | **-0.271962117** | -0.161528269 | 0.161528269 |
| **Vitamin B6 mg** | -0.003328839 | -0.190579917 | 0.190579917 |
| **Chlorum mg** | -0.045990772 | 0.014085992 | -0.014085992 |
| **Iodine mcg** | -0.09034727 | -0.101387615 | 0.101387615 |
| **Manganese mg** | 0.071833599 | -0.058970163 | 0.058970163 |
| **Pantothenic acid mg** | 0.094259395 | -0.05073502 | 0.05073502 |
| **Vitamin B12 mcg** | 0.087364899 | 0.060637653 | -0.060637653 |
