# Supplementary material for: Assessing the Influence of Vegan, Vegetarian and Omnivore Oriented Westernized Dietary Styles on Human Gut Microbiota: A Cross Sectional Study
Source: Front Microbiol. 2018 Mar 5;9:317. doi: 10.3389/fmicb.2018.00317 (PMC5844980; doi:10.3389/fmicb.2018.00317)
Supplement: Supplementary file 6 [file Data_Sheet_2.docx]

SUPPLEMENTARY RESULTS

*Correlation of anthropometric data, diet and microbiota*

When possible, Spearman’s correlation of microbiota profiles at genera level and anthropometric quantitative data (BMI, BLM, BFM) were investigated (data not shown). Higher levels of correlation were found between: BFM/BLM and *Enterobacter* abundance (rho= 0.3071756, significantly different from 0); BFM/BLM and the *Prevotellaceae* Family abundance (rho= 0.3445198, significantly different from 0); BMI and the *Prevotellaceae* Family abundance (rho= 0.4026421, significantly different from 0). Correlation between dietary profiles and anthropometric quantitative data (BMI, BLM, BFM) are reported in Table S4. Correlations between dietary profiles and genus-level microbiota abundances highlighted that *Subdoligranulum*, *Alistipes*, *Oxalobacter*, *Haemophilus*, *Flavobacterium* and *Odoribacter* are the genera showing significantly non-zero correlation with more than ten different micro-nutrients. Lastly, correlation between average caloric percentage of lipids, proteins and carbohydrates and Genus-level microbiota abundance results are graphically summarized in Figure S4.
