# Supplementary material for: Assessing the Influence of Vegan, Vegetarian and Omnivore Oriented Westernized Dietary Styles on Human Gut Microbiota: A Cross Sectional Study
Source: Front Microbiol. 2018 Mar 5;9:317. doi: 10.3389/fmicb.2018.00317 (PMC5844980; doi:10.3389/fmicb.2018.00317)

Supplementary Figure 1: Box plots of value of Body Fat Mass (BFM), Body Lean Mass (BLM), Body Mass Index (BMI) and Body Water Mass (BWM) of the participants by cohort (O=Omnivorous, V=Vegetarian, VG=Vegan)

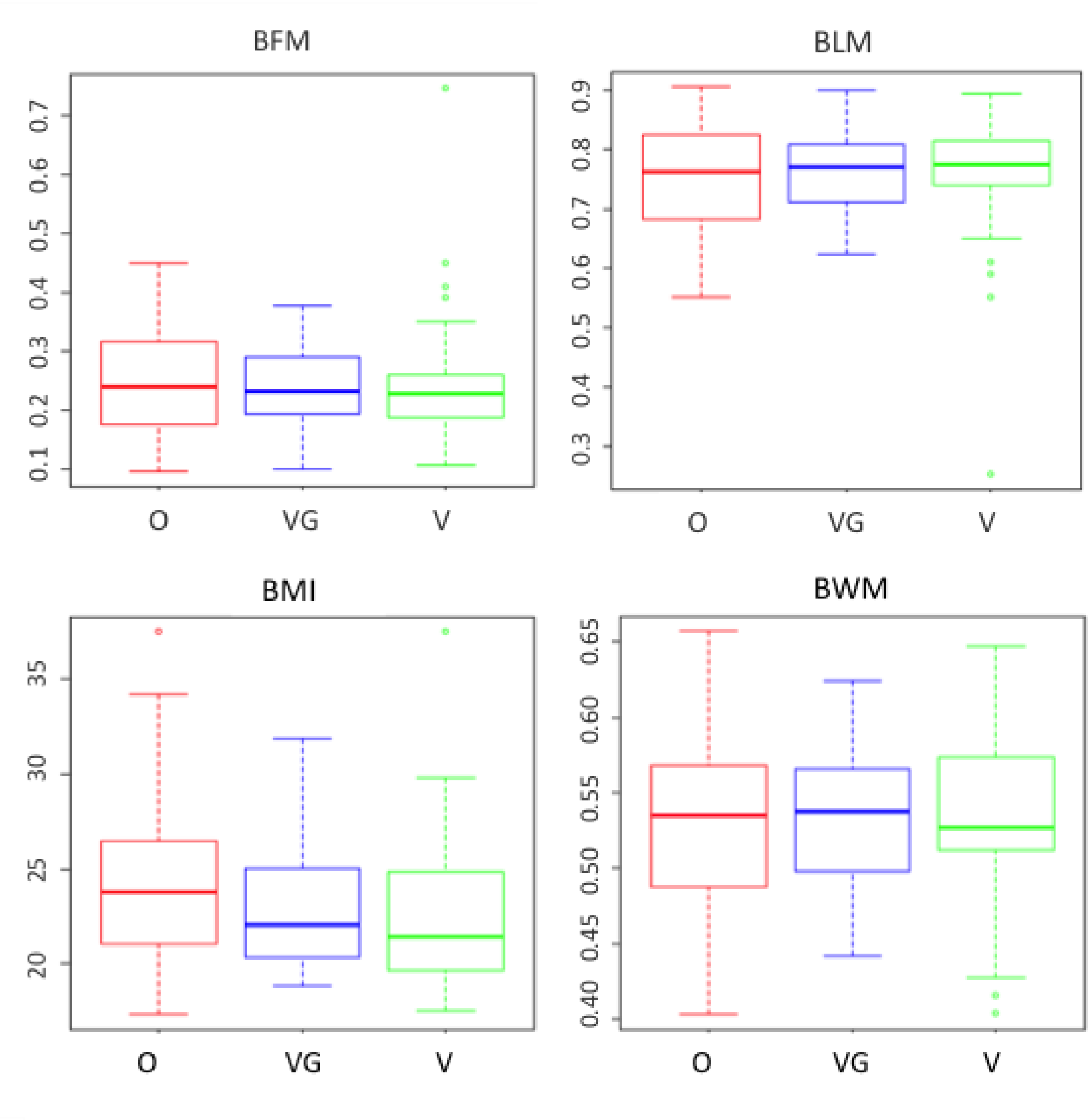

Supplement: Supplementary file 7 [file Image_1.pdf]
