# Supplementary material for: Assessing the Influence of Vegan, Vegetarian and Omnivore Oriented Westernized Dietary Styles on Human Gut Microbiota: A Cross Sectional Study
Source: Front Microbiol. 2018 Mar 5;9:317. doi: 10.3389/fmicb.2018.00317 (PMC5844980; doi:10.3389/fmicb.2018.00317)
Supplement: Supplementary file 8 [file Image_2.pdf]

Supplementary Figure 2: Random Forest on Dietary data Feature Ranking according to the mean decrease in accuracy

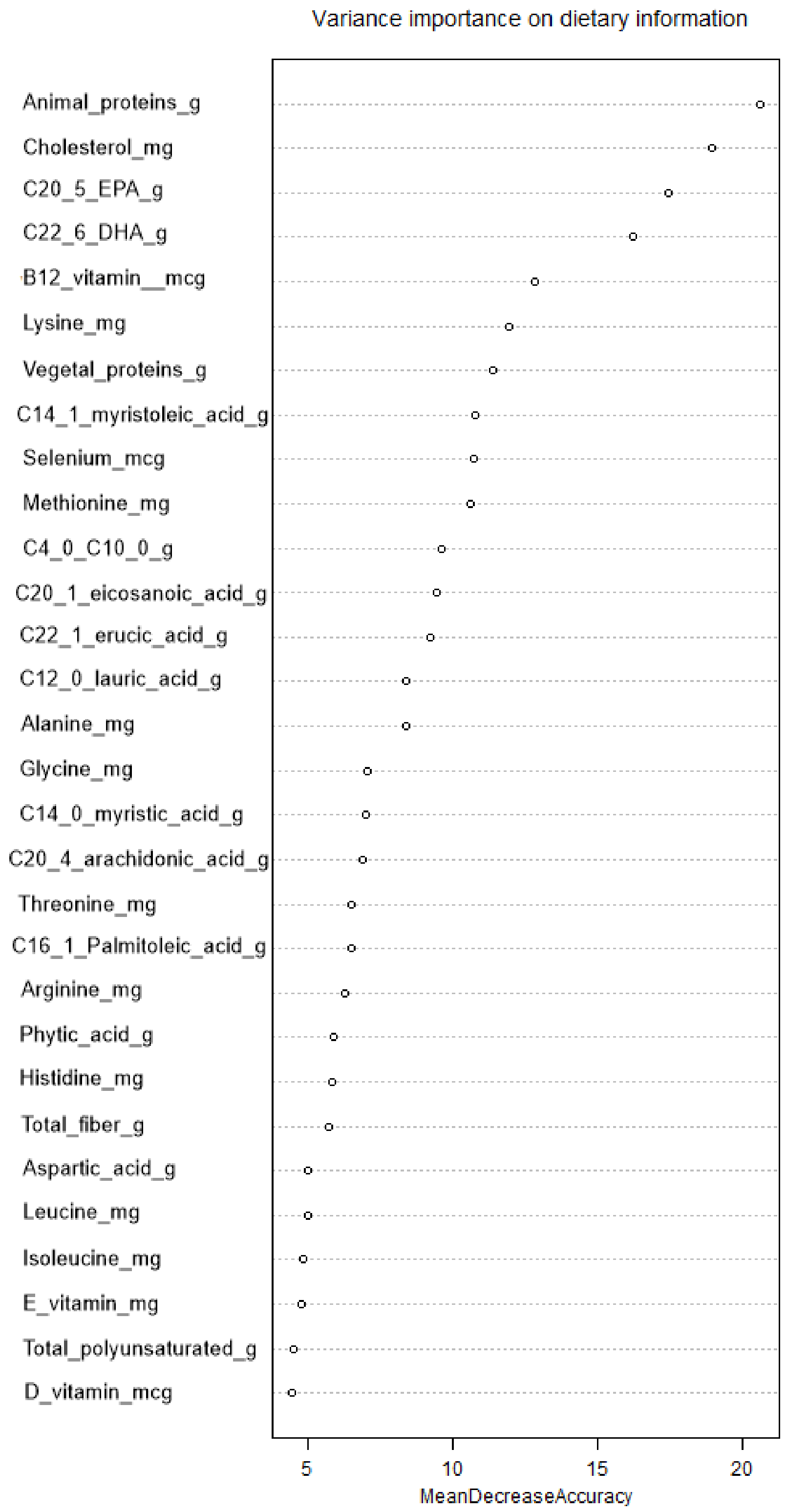
Variance importance on dietary information
Animal_proteins_g
Cholesterol_mg
C20_5_EPA_g
C22_6_DHA_g
B12_vitamin__mcg
Lysine_mg
Vegetal_proteins_g
C14_1_myristoleic_acid_g
Selenium_mcg
Methionine_mg
C4_0_C10_0_g
C20_1_eicosanoic_acid_g
C22_1_erucic_acid_g
C12_0_lauric_acid_g
Alanine_mg
Glycine_mg
C14_0_myristic_acid_g
C20_4_arachidonic_acid_g
Threonine_mg
C16_1_Palmitoleic_acid_g
Arginine_mg
Phytic_acid_g
Histidine_mg
Total_fiber_g
Aspartic_acid_g
Leucine_mg
Isoleucine_mg
E_vitamin_mg
Total_polyunsaturated_g
D_vitamin_mcg
5
10
15
20
MeanDecreaseAccuracy

5
