# Supplementary material for: Assessing the Influence of Vegan, Vegetarian and Omnivore Oriented Westernized Dietary Styles on Human Gut Microbiota: A Cross Sectional Study
Source: Front Microbiol. 2018 Mar 5;9:317. doi: 10.3389/fmicb.2018.00317 (PMC5844980; doi:10.3389/fmicb.2018.00317)

Supplementary Figure 3: Proportional abundance of the phyla found in each sample. X-axis represents the participants and the y-axis represents the proportion of counts assigned to each phylum.

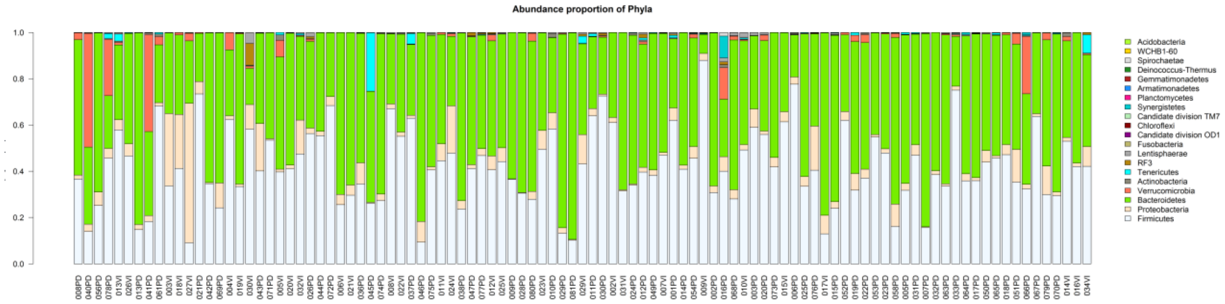

Supplement: Supplementary file 9 [file Image_3.pdf]
