# Supplementary material for: Assessing the Influence of Vegan, Vegetarian and Omnivore Oriented Westernized Dietary Styles on Human Gut Microbiota: A Cross Sectional Study
Source: Front Microbiol. 2018 Mar 5;9:317. doi: 10.3389/fmicb.2018.00317 (PMC5844980; doi:10.3389/fmicb.2018.00317)
Supplement: Supplementary file 10 [file Image_4.pdf]

Supplementary Figure 4: Heatmap of Spearman's correlation of average percentage of kcals and genera abundances

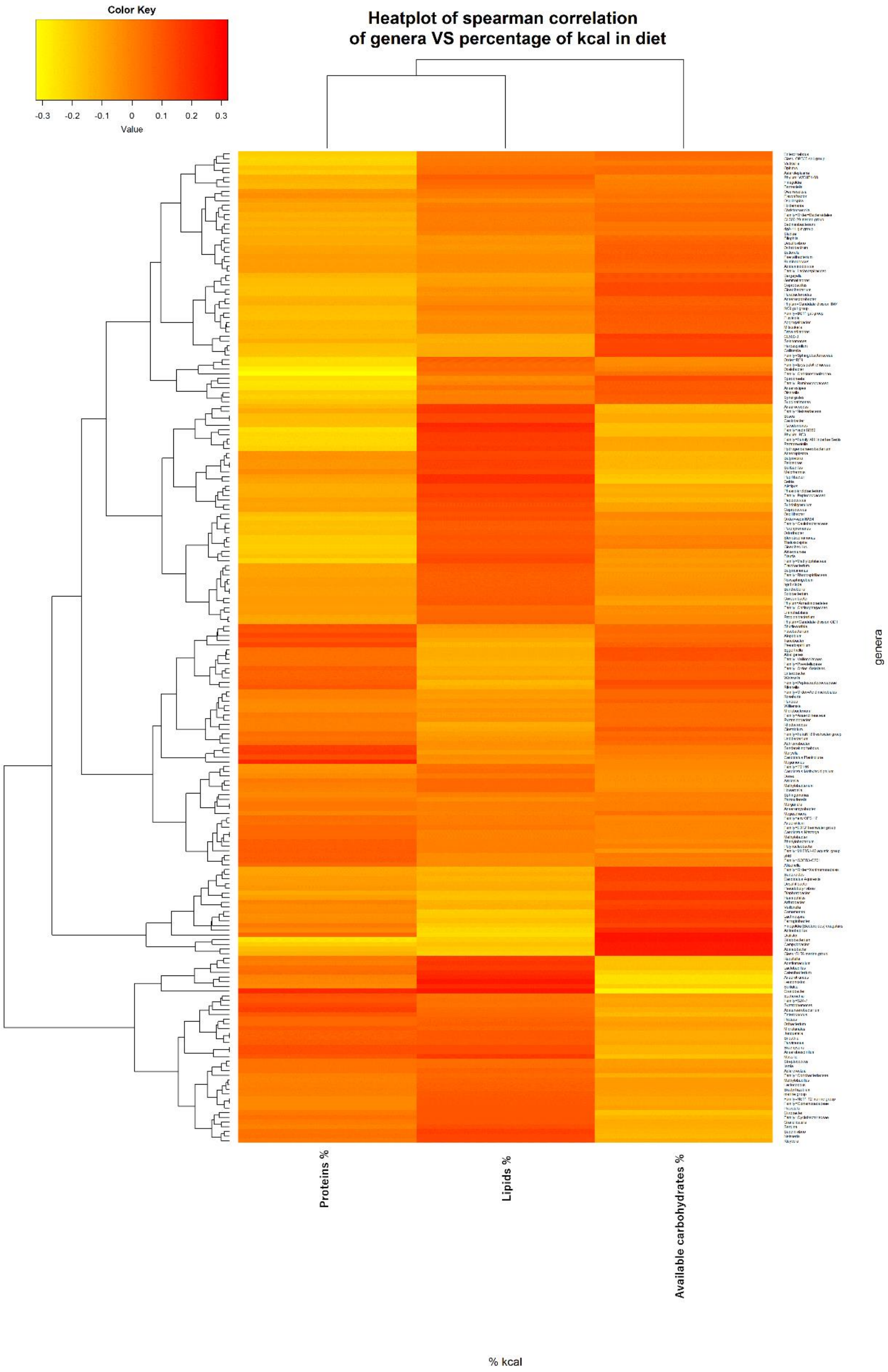
Color Key
-0.3 -0.2 -0.1 0 0.1 0.2 0.3
Value
Heatplot of spearman correlation
of genera VS percentage of kcal in diet
Proteins %
Lipids %
Available carbohydrates %
genera
% kcal
